# Application of distance transformation on parameter optimization of inverse planning in intensity‐modulated radiation therapy

**DOI:** 10.1120/jacmp.v9i2.2750

**Published:** 2008-04-16

**Authors:** Hui Yan, Fang‐Fang Yin

**Affiliations:** ^1^ Department of Radiation Oncology Duke University Medical Center Durham North Carolina U.S.A.

**Keywords:** Inverse planning, intensity‐modulated radiation therapy, parameter distribution, distance transformation

## Abstract

In inverse planning for intensity‐modulated radiation therapy (IMRT), the dose specification and related weighting factor of an objective function for involved organs is usually predefined by a single value and then iteratively optimized, subject to a set of dose—volume constraints. Because the actual dose distribution is essentially non‐uniform and considerably affected by the geometric shape and distribution of the anatomic structures involved, the spatial information regarding those structures should be incorporated such that the predefined parameter distribution is made to approach the clinically expected distribution. Ideally, these parameter distributions should be predefined on a voxel basis in a manual method. However, such an approach is too time‐consuming to be feasible in routine use.

In the present study, we developed a computer‐aided method to achieve the goal described above, producing a non‐uniform parameter distribution based on spatial information about the anatomic structures involved. The method consists of two steps:

- Use distance transformation technique to calculate the distance distribution of the structures.
- Based on the distance distribution, produce the parameter distribution via a conversion function guided by prior knowledge.

We use two simulated cases to examine the effectiveness of the method. The results indicate that application of a non‐uniform parameter distribution produced by distance transformation clearly improves dose‐sparing of critical organs without compromising dose coverage of the planning target.

PACS numbers: 87.53.Jw

## I. INTRODUCTION

Inverse treatment planning for intensity‐modulated radiation therapy (IMRT) involves optimization of an intensity map by application of an objective function to obtain an ideal dose distribution. The ideal dose distribution usually requires that doses for the critical organs and surrounding normal tissue fall below tolerated doses, and that the dose for the tumor meets the minimum dose prescribed by the physician. Achieving this goal requires refinement of a combination of parameters predefined for the objective function of inverse planning. In recent years, several parameter optimization methods have been developed to improve the effectiveness (dose conformity, uniformity) and efficiency (delivery time, number of segments, subsequent number of monitor units) of IMRT.^(^ ^1^ ^–^ ^12^ ^)^

Initially, a single value is used to represent the parameter distribution of an objective function. After the dose distribution is calculated, the parameter distribution is iteratively modified based on various optimization techniques subject to various dose constraints.^(^ ^3^ ^–^ ^5^ ^)^ Currently, parameter optimization techniques are the focus of considerable research efforts, but little attention is being paid to the optimization of the predefined parameters of an objective function in inverse planning.

In conventional inverse planning, the initial calculation of dose distribution is followed by selection of the predefined parameters of an objective function based explicitly on the dose—volume constraint^(^ ^2^ ^–^ ^4^ ^)^ or equivalent uniform dose^(5)^. No direct method exists for specifying the distributions of those predefined parameters on a voxel basis. The technique of distance transformation makes it possible to automatically produce the parameter distributions based on the geometric distributions of the anatomic structures involved. Accordingly, the physician's treatment intent can be properly incorporated.

In the present study, we propose a computer‐based method to incorporate the spatial information from anatomic structures into the parameter specification of the objective function in inverse planning. On a voxel basis, parameter specifications are determined by their spatial location and distance from the important anatomic structures. A non‐uniform parameter distribution approximating the clinically expected distribution and featuring higher priority for the voxels around the target volume and lower priority for the voxels away from the target volume are then automatically produced. Briefly, the method is implemented in two steps:

- The distance distribution of the entire volume of an anatomic structure is calculated based on the distance transformation technique.
- The parameter distribution is produced from the distance distribution via a conversion function guided by the prior knowledge of the human planner.

In a preliminary evaluation, we examined the parameter distribution of the weighting factor for critical organs and normal tissue of an objective function determined using our method, and we performed fluence optimization on an inverse planning system developed in‐house at our institution. Simulated cases were used to examine two types of non‐uniform parameter distributions. For comparison purposes, we also investigated the results of uniform parameter distributions for the same cases. We then compared the resulting dose distributions and dose—volume histograms (DVHs) for the three types of parameter distributions (two non‐uniform distributions and one uniform distribution). The advantages and disadvantages of the computer‐based method for parameter specification are discussed.

## II. MATERIALS AND METHODS

### A. Introduction of distance transformation

In many digital image processing applications, the distance from certain feature elements to non‐feature elements is an important factor considered in classifying the elements of the non‐feature volume into various categories.^(^ ^11^ ^–^ ^21^ ^)^ A “distance transformation” (DT) is an operation that converts a binary picture consisting of feature and non‐feature elements into a picture in which each non‐feature element is assigned a value that approximates the distance to the nearest feature element. Several two‐dimensional DTs have been developed.^(13)^ Theoretically, all can be generalized to higher dimensions. Among them, the chamfer DT is the fastest and simplest to implement, and it performs accurately in approximating to real distance.^(11)^

In the simplest case of one‐dimensional chamfer DT, the elements in a line are initially set to two values: 0 for feature elements and ∞ for non‐feature elements. The algorithm consists of two “passes” along the line. The first pass, called the forward pass, changes the values of the elements as follows: For i=2,3,…,M,

(1) $$v_{i}^{1}=\text{minimum}(v_{i}^{0},v_{i-1}^{1}+1),$$

where $v_{i}^{0}$ is the initial value and $v_{i}^{1}$ is the new value of element *i*, and *M* is the number of elements.

After the forward pass, each element has a value equal to its distance to the nearest feature element to its left. The second pass, called the backward pass, changes the values as follows: For i=M−1,M−2,…,1,

(2) $$v_{i}^{2}=\text{minimum}(v_{i}^{1},v_{i+1}^{2}+1),$$

where $v_{i}^{1}$ and $v_{i}^{2}$ are, respectively, the previous and new value of element *i*.

The backward pass measures a non‐feature element's nearest distance to the feature element to its right. After the backward pass, all non‐feature elements show the correct distance to the nearest feature element.

**Figure 1:**
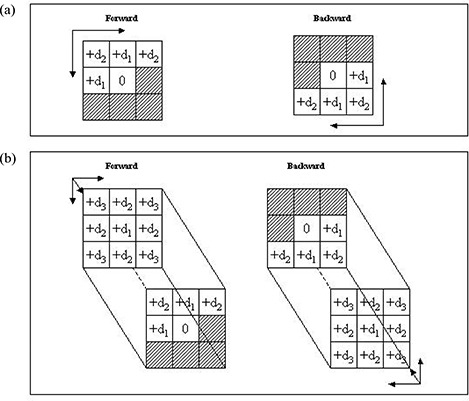
Illustration of mask elements for (a) two‐dimensional and (b) three‐dimensional distance (*d*) transformation.

By extension, in cases of two‐dimensional transformation, each element has two types of neighbors: horizontal or vertical (n=4), and diagonal (n=4) [Fig. 1(a)]. The two passes start from the upper left‐hand corner and the bottom right‐hand corner and move in opposite directions. In cases of three‐dimensional transformation, each element has three types of neighbors [Fig. 1(b)]: voxels in a plane (n=6), neighbors joined by a line (n=12), and neighbors joined by only a point (n=8). The two passes start from the top front left‐hand corner of the volume and bottom back right‐hand corner of the volume, and move in opposite directions. (More details of two‐dimensional and three‐dimensional DTs can be found in Appendix I.)

### B. Objective function

In the present study, the anatomic structures of an inverse plan are briefly grouped into three categories: target volume (TV), critical organ (CO), and normal tissue (NT). An iterative gradient algorithm is used to calculate the intensity spectrum, *x*. The objective function is

(3) $$f(x)\sum_{i}\sum_{j}\sum_{k}w_{ijk}{\left(d_{ijk}-p_{ijk}\right)}^{2},$$

where $d_{ijk}=\sum_{n=1}^{N}A_{n,ijk}x_{n}$ is the calculated dose at voxel $(i,j,k)A_{n,ijk}$ is a non‐negative constant (the relative dose coefficient—that is, dose per unit intensity of pencil beam *n*), and $w_{ijk}$ and $p_{ijk}$ are, respectively, the weighting factor and dose prescription, defined as

(4) $$\begin{array}{l} w_{ijk}=\{\begin{array}{l} W_{NT}+W_{TV},\;\;\;\;\;if\;\;\;\;\;(i,j,k)\in \Omega_{TV}\\ W_{NT}+W_{CO},\;\;\;\;\;if\;\;\;\;\;(i,j,k)\in \Omega_{CO}\\ W_{NT},\;\;\;\;\;if\;\;\;\;\;\;(i,j,k)\in \Omega_{NT} \end{array}\\ \;,\text{and} \end{array}$$

(5) $$\begin{array}{l} p_{ijk}=\{\begin{array}{l} P_{TV},\;\;\;\;\;if\;\;\;\;\;(i,j,k)\in \Omega_{TV}\\ P_{CO},\;\;\;\;\;if\;\;\;\;\;(i,j,k)\in \Omega_{CO}\\ P_{NT},\;\;\;\;\;if\;\;\;\;\;\;(i,j,k)\in \Omega_{NT} \end{array}\\ \;, \end{array}$$

where $\Omega_{TV},\Omega_{CO}$, and $\Omega_{NT}$ denote, respectively, the regions of the target volume, critical organs, and normal tissue; and $W_{TV}+W_{NT},W_{CO}+W_{NT}$, and $W_{NT}$ are the weighting factors specified for, respectively, the target volume, critical organs, and normal tissue.

The minimization of the objective function under the constraint $x_{n}\geq 0$ can be written in the form

(6) $$\begin{array}{l} \underset{x}{\min}\left\{f(x)\right\}\\ \text{subject}\;\text{to}\;x_{n}\geq 0,\forall n. \end{array}$$

The fast monotonic descent (FMD) method developed by Li and Yin^(6)^ is used to minimize this objective function. The FMD method uses an optimal step length to guarantee fast and monotonic convergence to the global minimum of the given objective function. The final intensity map, $x=(x1,x2,\ldots ,x_{N})$, is achieved when the FMD converges.

### C. Non‐uniform parameter distribution

**Figure 2:**
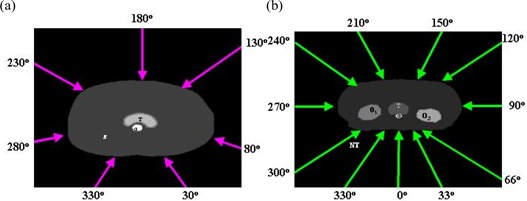
Arrangement of beam angles in (a) case 1 and (b) case 2.

**Figure 3:**
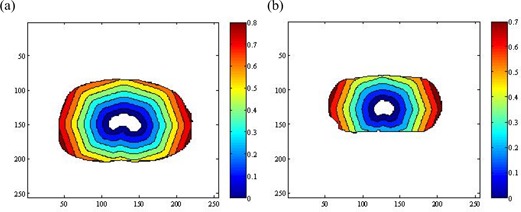
Distance distributions achieved on the isocenter slice of (a) case 1 and (b) case 2.

Fig. 2(a) and Fig. 2(b) show the spatial distributions of the anatomic structures and beam arrangement in two simulated cases. The target volume region was defined as the feature volume, and the joint regions of the critical organs and normal tissue were defined as the non‐feature volume. Application of the three‐dimensional DT generated the distance distribution for the critical organs and normal tissue, which was then scaled to a range [0, 1] at its maximum. Fig. 3 shows the distance distributions at the isocenter slice for both cases. The target volume region is white because its value is automatically set to 0.

Having generated the distance distributions shown in Fig. 3, various methods can be used to derive the distribution of the weighting factor. Here, we employed two functions to convert the distance distribution to the distribution of the weighting factor.

The first function implements a linear conversion of the distance distribution:

(7) $$w_{ijk}^{B}=(1-d_{ijk})*W_{NT},$$

where $w_{ijk}^{B}$ and $d_{ijk}$ are the weighting factor and the value of distance distribution for a voxel (*i, j, k*) and $W_{NT}$ is the constant defined in equation 5. The weighting factor generated in this way for the element combining the critical organs and normal tissue is in inverse proportion to its distance from the target volume and changes evenly. The weighting factor produced by equation 7 for the target volume is a single value, $W_{NT}$. The final value of the weighting factor is calculated from equation 5:

(8) $$\begin{array}{l} w_{ijk}=\{\begin{array}{l} w_{ijk}^{B}+W_{TV},\;\;\;\;\;if\;\;\;\;(i,j,k)\in \Omega_{TV}\\ w_{ijk}^{B}+W_{CO},\;\;\;\;\;if\;\;\;\;(i,j,k)\in \Omega_{CO}\\ w_{ijk}^{B}\;\;\;\;\;\;\;\;\;\;\;,\;\;\;\;\;if\;\;\;\;(i,j,k)\in \Omega_{NT} \end{array}\\ \;. \end{array}$$

**Figure 4:**
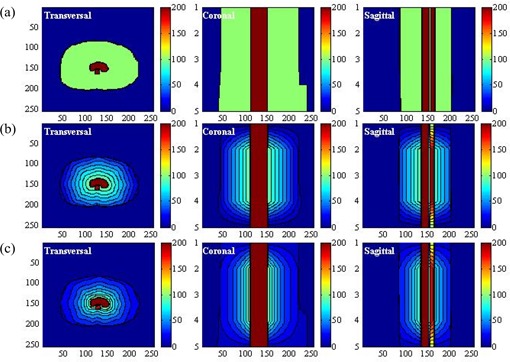
(a) Display of the uniform parameter distribution made by the conventional method in the transversal, coronal, and sagittal planes in case 1. (b) Display of the non‐uniform parameter distribution generated by the linear function in the transversal, coronal, and sagittal planes in case 1. (c) Display of the non‐uniform parameter distribution generated by the exponential function in the transversal, coronal, and sagittal planes in case 1.

As a demonstration, Figs. 4(a) (case 1) and 5(a) (case 2) show the conventional parameter distributions in the transversal, coronal, and sagittal planes as defined by equation 5. By comparison, Figs. 4(b)) (case 1) and 5(b) (case 2) show the distribution of the weighting factor as defined by equation 7. The weighting factor of the voxels in the combined region of critical organs and normal tissue can be seen to be enhanced as they approach the target volume.

**Figure 5:**
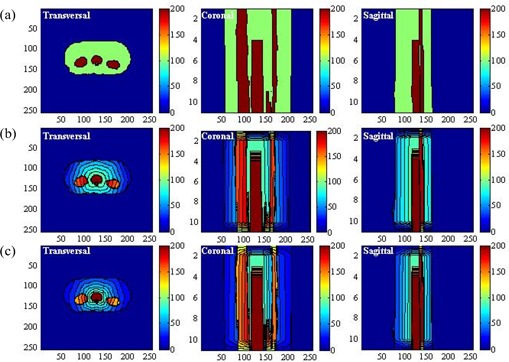
(a) Display of the uniform parameter distribution made by the conventional method in the transversal, coronal, and sagittal planes in case 2. (b) Display of the non‐uniform parameter distribution generated by the linear function in the transversal, coronal, and sagittal planes in case 2. (c) Display of the non‐uniform parameter distribution generated by the exponential function in the transversal, coronal, and sagittal planes in case 2.

The second function is an exponential function designed to implement a non‐linear conversion of the distance distribution:

(9) $$w_{ijk}^{B}=\exp(-2d_{ijk})*W_{NT}.$$

Here, $w_{ijk}^{B}$ and $d_{ijk}$ are the base weighting factor and distance value for voxel (*i, j, k*) and $W_{NT}$ is same constant defined earlier. As a result, the weighting factor of the voxel in the region of combined critical organs and normal tissue is in inverse proportion to the distance distribution and increases sharply as the voxel approaches the target volume. For situations in which a higher dose is prohibited for the critical organs in the area surrounding the target volume, the extra protection provided is needed, and this parameter distribution will be a better choice.

When the base distribution of the weighting factor is reached, equation 8 can generate the final distribution. Figs. 4(c) and 5(c) show that distribution in the three orthogonal planes for the two cases under consideration. Comparing the conventional parameter distribution and the distribution generated by the linear function of equation 8, the distribution generated by equation 9 can be seen to assign more priority to the voxels of the critical organs and normal tissue in proximity to the target volume.

### D. Evaluation method

We used two simulated cases to evaluate the application of DT in inverse planning optimization:

- In the first case, one critical organ is surrounded by target volumes, and 7 coplanar beams are arranged as shown in Fig. 2(a).
- The second case involves three critical organs, one of which is adjacent to the target volume. (The computed tomography data for the second case came from an actual treated patient.) As shown in Fig. 2(b), 11 coplanar beams are used. For simplicity, the dose was approximated by the primary 6‐MV photon beam only. Beam divergence was included in the calculation.

For both cases, the dose prescription for the tumor, critical organs, and normal tissue was set to constant values of $P_{TV}=100$ Gy, $P_{CO}=20$ Gy, and $P_{NT}=50$ Gy respectively. The weighting factors for the tumor and critical organs were set to $W_{TV}=100$ and $W_{CO}=100$ respectively. The weighting factor for the normal tissue was set to $W_{NT}=100$.

We next compared the performance of the non‐uniform parameter distributions generated by the computer‐aided method with the uniform parameter distribution. In the conventional plan (scheme 1), the uniform parameter distribution shown in Figs. 4(a) and 5(a) was used. In the second and third plans (schemes 2 and 3), the non‐uniform parameter distributions shown in Figs. 4(b),(c) and 5(b,c), as generated by equations 7 and 9 were used. We used isodose distributions and dose—volume histograms to evaluate the performance of the three schemes. To verify the effectiveness of the non‐uniform parameter distribution with various magnitudes of $W_{NT}$, we tested 5 values of $W_{NT}$ (20, 40, 60, 80, 100). The calculated dose for each plan was normalized by its dose at isocenter. We evaluated the doses at the 90% and 80% volumes for the target volume [$V^{TV}$(90) and $V^{TV}$(80)], the doses at the 60% and 40% volumes for the critical organs [$V^{CO}$(60) and $V^{CO}$(40)], and the doses at the 50% and 30% volumes for the normal tissue ([$V^{NT}$(50) and $V^{NT}$(30)] and summarized the results. Using the same setup, we compared the differences between the doses in the three schemes.

## III. RESULTS

**Figure 6:**
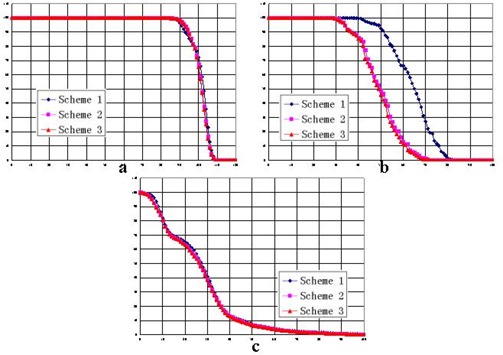
Dose—volume histograms of the three schemes in case 1 for (a) the target volume, (b) the critical organ, and (c) the normal tissue.

**Figure 7:**
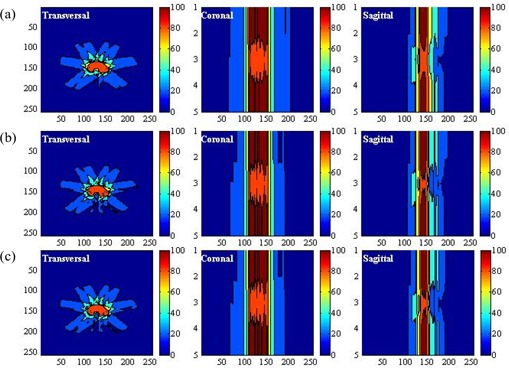
Display of the dose distributions in the transversal, coronal, and sagittal planes in case 1 for (a) scheme 1, (b) scheme 2, and (c) scheme 3.

For case 1, with $W^{NT}$ set to 100, Fig. 6(a–c) compares the DVHs achieved by the three schemes for the target volume, critical organs, and normal tissue respectively. Fig. 7(a–c) shows the isodose distributions in the transversal, coronal, and sagittal planes for schemes 1, 2, and 3 respectively. Compared with the DVH achieved by scheme 1, the dose to the critical organ was significantly reduced (by approximately 20%) with the application of non‐uniform parameter distribution.

**Figure 8:**
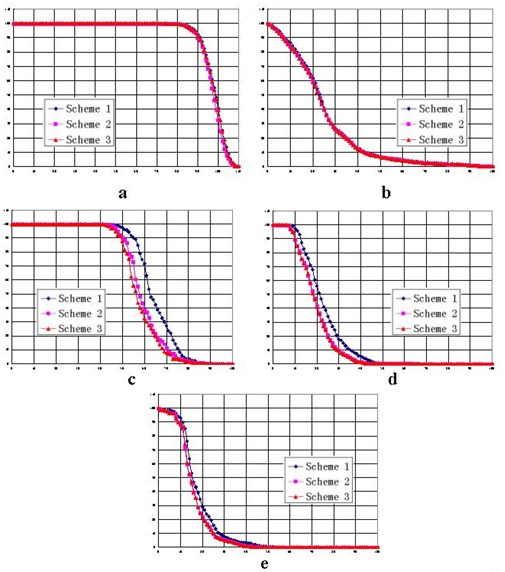
Dose—volume histograms of the three schemes in case 2, with *W* set to 20, for (a) the target volume, (b) the normal tissue, (c) critical organ 1, (d) critical organ 2, and (e) critical organ 3.

**Figure 9:**
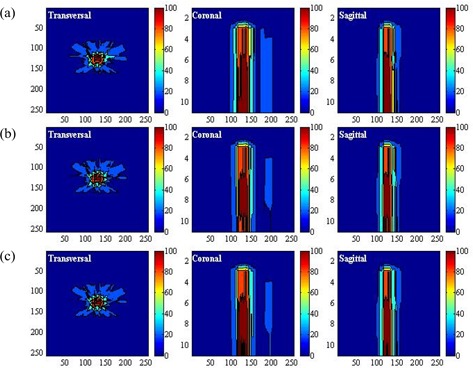
Display of the dose distributions in the transversal, coronal, and sagittal planes in case 2 for (a) scheme 1, (b) scheme 2, and (c) scheme 3.

For case 2, Figs. 8 and 9 show the DVHs and isodose distributions achieved by the three schemes. Improved dose sparing for the three critical organs is observed. The improvement is approximately 10% for critical organ 1, and 5% and 2% for critical organs 2 and 3. The dose distributions achieved by schemes 2 and 3 are similar. Dose‐sparing of critical organ 1 in schemes 2 and 3 is apparently improved as compared with the sparing achieved by scheme 1.

A comparison of the three schemes, varying the magnitude of the weighting factor, shows that the dose‐sparing of critical organs achieved by schemes 2 and 3 is always superior to the sparing presented in scheme 1.

For case 1, Table 1 summarizes the doses at the specific percentage volumes for various values of $W^{NT}$. Table 2 shows the calculated differences between the three schemes. On average, 15% and 16% improvements of dose‐sparing to the critical organ were achieved by schemes 2 and 3 respectively. No apparent degradation of tumor dose was observed.

For the clinical case (case 2), Table 3 summarizes the doses at the specific percentage volumes with various values of $W^{NT}$. Table 4 shows the calculated dose differences between the three schemes. On average, schemes 2 and 3 respectively achieved 5% and 7% improvements of dose‐sparing to critical organ 1. For the other two critical organs, a dose reduction of about 2% was observed, and a comparable tumor dose was obtained.

As shown in Tables 2 and 4, the differences between the doses from schemes 2 and 3 are smaller than the differences between scheme 3 and scheme 1 or those between scheme 2 and scheme 1. Scheme 3 shows a slight advantage over scheme 2. As Figs. 6 and 8 show, scheme 3 achieved the best dose‐sparing of the critical organs. The superiority of scheme 3 is also demonstrated by the isodose distributions (Figs. 7 and 9).

As compared with the dose distributions in scheme 1, the dose distributions in schemes 2 and 3 show an evident improvement of dose‐sparing for the critical organs. As $W^{NT}$ increases from 20 to 100, that improvement remains consistent (Tables 2 and 4). The difference between schemes 2 and 3 is smaller, with an average difference of 1% in case 1 and 2% in case 2.

## IV. DISCUSSION

Application of non‐uniform parameter distribution, with incorporation of spatial information from the structures involved, improved the optimization results of inverse planning. As a consequence, dose‐sparing for critical organs in proximity to the target volume was improved. Two simulated cases verified the effectiveness of this computer‐aided parameter distribution. In case 1 (with a single critical organ), the dose to the critical organ was significantly reduced when non‐uniform parameter distributions were applied. In case 2 (with three critical organs), the dose to critical organ 1, closest to the tumor, was apparently reduced. A smaller improvement of dose sparing was observed for the other two critical organs, at greater distances from the target volume. The implication is that non‐uniform parameter distribution is more important for critical organs closer to the target volume. Both cases indicated that, with application of the non‐uniform parameter distribution, dose‐sparing of critical organs in proximity to the target volume could be improved.

**Table 1:** Doses for specific percentage volumes (V) in case 1

*Percentage volume (%)*	*Scheme 1*					*Dose (%) Scheme 2*					*Scheme 3*				
	$W_{NT}=20$	$W_{NT}=40$	$W_{NT}=60$	$W_{NT}=80$	$W_{NT}=100$	$W_{NT}=20$	$W_{NT}=40$	$W_{NT}=60$	$W_{NT}=80$	$W_{NT}=100$	$W_{NT}=20$	$W_{NT}=40$	$W_{NT}=60$	$W_{NT}=80$	$W_{NT}=100$
$V^{TV}(90)$	95	94	94	94	93	95	95	95	95	95	95	95	95	95	95
$V^{TV}(80)$	97	98	98	98	98	97	97	98	98	98	96	97	98	98	98
$V^{CO}(60)$	44	53	58	61	63	31	38	42	45	47	31	37	42	45	47
$V^{CO}(40)$	50	59	63	66	68	35	42	47	51	53	34	41	46	50	52
$V^{NT}(50)$	28	28	28	28	28	26	27	27	27	27	26	26	27	27	27
$V^{NT}(30)$	34	34	34	34	33	33	33	33	33	33	33	33	33	33	33

W=weighting factor; NT=normal tissue; TV=target volume; CO=critical organ

**Table 2:** Dose difference for specific percentage volumes (V) between the three schemes in case 1

*Percentage volume (%)*	*Scheme 2 – Scheme 1*					*Dose difference (%) Scheme 3 – Scheme 1*					*Scheme 3 – Scheme 2*				
	$W_{NT}=20$	$W_{NT}=40$	$W_{NT}=60$	$W_{NT}=80$	$W_{NT}=100$	$W_{NT}=20$	$W_{NT}=40$	$W_{NT}=60$	$W_{NT}=80$	$W_{NT}=100$	$W_{NT}=20$	$W_{NT}=40$	$W_{NT}=60$	$W_{NT}=80$	$W_{NT}=100$
$V^{TV}(90)$	0	1	1	1	2	0	1	1	1	2	0	0	0	0	0
$V^{TV}(80)$	0	−1	0	0	0	−1	−1	0	0	0	−1	0	0	0	0
$V^{CO}(60)$	−13	−15	−16	−16	−16	−13	−16	−16	−16	−16	0	−1	0	0	0
$V^{CO}(40)$	−15	−17	−16	−15	−15	−16	−18	−17	−16	−16	−1	−1	−1	−1	−1
$V^{NT}(50)$	−2	−1	−1	−1	−1	−2	−2	−1	−1	−1	0	−1	0	0	0
$V^{NT}(30)$	−1	−1	−1	−1	0	−1	−1	−1	−1	0	0	0	0	0	0

W=weighting factor; NT=normal tissue; TV=target volume; CO=critical organ

**Table 3:** Doses for specific percentage volumes (V) in case 2

*Percentage volume (%)*	*Scheme 1*					*Dose (%) Scheme 2*					*Scheme 3*				
	$W_{NT}=20$	$W_{NT}=40$	$W_{NT}=60$	$W_{NT}=80$	$W_{NT}=100$	$W_{NT}=20$	$W_{NT}=40$	$W_{NT}=60$	$W_{NT}=80$	$W_{NT}=100$	$W_{NT}=20$	$W_{NT}=40$	$W_{NT}=60$	$W_{NT}=80$	$W_{NT}=100$
$V^{TV}(90)$	93	93	92	92	92	92	92	91	91	90	92	92	92	91	91
$V^{TV}(80)$	96	96	95	94	94	96	95	94	94	93	96	95	95	94	94
$V^{CO-1}\left(60\right)$	46	54	58	60	62	41	49	52	55	57	39	46	51	53	55
$V^{CO-1}\left(40\right)$	52	59	63	65	66	47	54	57	59	60	44	51	55	57	59
$V^{CO-2}\left(60\right)$	19	19	19	19	20	18	18	17	17	17	18	18	17	17	17
$V^{CO-2}\left(40\right)$	22	23	23	24	24	20	20	20	21	21	20	20	20	21	21
$V^{CO-3}\left(60\right)$	15	15	15	15	15	15	14	14	14	13	16	15	14	14	14
$V^{CO-3}\left(40\right)$	18	18	18	18	18	18	17	17	17	16	18	18	17	17	17
$V^{NT}(50)$	24	24	24	24	24	23	23	23	23	23	23	23	23	23	23
$V^{NT}(30)$	30	29	29	29	29	28	29	29	29	29	28	28	29	29	29

W=weighting factor; NT=normal tissue; TV=target volume; CO=critical organ

**Table 4:** Dose difference for specific percentage volumes (V) between the three schemes in case 2

*Percentage volume (%)*	*Scheme 2 – Scheme 1*					*Dose difference (%) Scheme 3 – Scheme 1*					*Scheme 3 – Scheme 2*				
$W_{NT}=20$	$W_{NT}=40$	$W_{NT}=60$	$W_{NT}=80$	$W_{NT}=100$	$W_{NT}=20$	$W_{NT}=40$	$W_{NT}=60$	$W_{NT}=80$	$W_{NT}=100$	$W_{NT}=20$	$W_{NT}=40$	$W_{NT}=60$	$W_{NT}=80$	$W_{NT}=100$
$V^{TV}(90)$	−1	−1	−1	−1	−2	−1	−1	0	−1	−1	0	0	1	0	1
$V^{TV}(80)$	0	−1	−1	0	−1	0	−1	0	0	0	0	0	1	0	1
$V^{CO-1}\left(60\right)$	−5	−5	−6	−5	−5	−7	−8	−7	−7	−7	−2	−3	−1	−2	−2
$V^{CO-1}\left(40\right)$	−5	−5	−6	−6	−6	−8	−8	−8	−8	−7	−3	−3	−2	−2	−1
$V^{CO-2}\left(60\right)$	−1	−1	−2	−2	−3	−1	−1	−2	−2	−3	0	0	0	0	0
$V^{CO-2}\left(40\right)$	−2	−3	−3	−3	−3	−2	−3	−3	−3	−3	0	0	0	0	0
$V^{CO-3}\left(60\right)$	0	−1	−1	−1	−2	1	0	−1	−1	−1	1	1	0	0	1
$V^{CO-3}\left(40\right)$	0	−1	−1	−1	−2	0	0	−1	−1	−1	0	1	0	0	1
$V^{NT}(50)$	−1	−1	−1	−1	−1	−1	−1	−1	−1	−1	0	0	0	0	0
$V^{NT}(30)$	−2	−1	−1	−1	−2	−2	−1	0	0	0	0	−1	0	0	0

W=weighting factor; NT=normal tissue; TV=target volume; CO=critical organ

A comparison of schemes using varying values of weighting factor $W_{NT}$ showed better dose‐sparing of critical organs with schemes 3 and 2, with scheme 3 being the best of the two. That finding implies that the non‐uniform parameter distribution generated by the exponential function provides better dose sparing of critical organs.

The functions to implement a conversion of distance distribution depend on prior knowledge concerning the expected dose distribution of a treatment plan. Intuitively, the distance variables in equations 7 and 9 can be inversely weighted by the expected gradient of dose distribution around the target. Developing a suitable conversion function for a desired parameter distribution will require a more rigorous method based on the physician's treatment intent and the planner's clinical experience. Our next goal is to translate this technique into a useful clinical tool.

The application of a non‐uniform parameter distribution is not limited to the weighting factor demonstrated in the present study. The technique could be a powerful addition to other parameter optimization techniques based on dose—volume constraints or equivalent uniform doses. For more challenging clinical cases, optimization of the weighting factor alone is not sufficient; other parameters to be predefined in an objective function should be similarly optimized. However, because specification of certain parameters (such as dose prescription) is more closely related to the treatment intent than to the spatial distribution of anatomic structures, caution in the use of this technique would be required. Because the assumption of parameter distribution can be implemented in a conversion function as described earlier (see II.C, “Nonuniform parameter distribution”), incorporating those factors into the conversion function to create a more complicated parameter distribution that satisfies clinical requirements would be entirely possible.

## V. CONCLUSION

We developed a computer‐aided method to produce a non‐uniform parameter distribution for the parameter predefined in the objective function of inverse planning, thereby incorporating into the treatment plan spatial information about the anatomic structures involved. We examined the effectiveness of non‐uniform parameter distribution produced by DT technique regarding improvement of dose quality in a treatment plan, and we verified the results in two simulated cases. Our findings indicate that our method is capable of improving dose‐sparing for critical organs and normal tissue without compromising dose coverage to the planning target volume. The current method based on DT is useful not only in parameter specification in the early stages of inverse planning optimization, but also in parameter adjustment during IMRT optimization. It can also act as a valuable addition to existing inverse planning optimization techniques.

## ACKNOWLEDGMENT

We highly appreciate the extensive editing support received from Mrs. Jane Hoppenwoth for this manuscript.

## Appendix

In two‐dimensional chamfer distance transformation (DT), each element has two kinds of neighbors [Fig. 1(a)]: horizontal or vertical neighbors (n=4), and diagonal neighbors (n=4). Here, the distance between horizontal (or vertical) neighbors is denoted $d_{1}$, and the distance between diagonal neighbors is denoted $d_{2}$.

If the local distances $d_{1}$ and $d_{2}$ are set to their correct values, $d_{1}=1$ and $d_{2}=2^{1/2}$, the computed distance becomes the Euclidean length of the shortest path between two elements. A good integer approximation of the optimal values can be found by multiplying $d_{1}$ and $d_{2}$ by 3, which gives the integer value $d_{1}=3$ and $d_{2}=4$.

As in the one‐dimensional case, two passes over the picture are necessary [Fig. 1(a)]. In the forward pass, the mask starts in the upper left‐hand corner of the picture, moves from the left to the right corner, and from the top to the bottom row. In the backward pass, the mask starts in the lower right‐hand corner, moves from right to left, and from bottom to top. The local distances $d_{1}$ and $d_{2}$ in the mask pixels are added to the pixel values in the picture, and the new value of each pixel is the minimum of the five sums.

Extension of DT from two dimensions to three dimensions is relatively straightforward, except for the rapidly increasing quantity of data. Each voxel now has 26 neighbors of three different kinds [Fig. 1(b))]: neighbors in a plane (n=6), neighbors joined by a line (n=12), and neighbors joined by just one point (n=8).

The local distances to the different kinds of neighbors are denoted $d_{1}$, $d_{2}$, and $d_{3}$. By setting $d_{1}=1$, $d_{2}=2^{1/2}$, and $d_{3}=3^{1/2}$, the chamfer distance becomes the Euclidean length of the shortest path between two voxels. A good integer approximation of the optimal local distance is $d_{1}=3$, $d_{2}=4$, and $d_{3}=5$ by multiplying $d_{1}$, $d_{2}$, and $d_{3}$ by 3.

Fig. 1(b) demonstrates the 3‐dimensional chamfer algorithm. Two passes over the volume are necessary. In the forward pass, the mask is moved over the volume from left to right, top to bottom, and front to back. In the backward pass, the mask is moved in the opposite direction. In each position, the total local distance in each mask voxel and the value of the voxel it covers are computed, and the new value of the voxel is the minimum of those sums. The local distances $d_{1}$, $d_{2}$, and $d_{3}$ in the mask voxels are added to the voxel values in the volume, and the new value of the voxel is the minimum of the 14 sums.
